# Supplementary material for: Comprehensive Annotation and Functional Exploration of MicroRNAs in Lettuce
Source: Front Plant Sci. 2021 Dec 24;12:781836. doi: 10.3389/fpls.2021.781836 (PMC8739914; doi:10.3389/fpls.2021.781836)
Supplement: Supplementary file 2 [file Data_Sheet_8.ZIP › GSM2230747.plot/Lsa-miR396c_Lsat_1_v5_gn_5_72841.1_2120_TPlot.pdf]

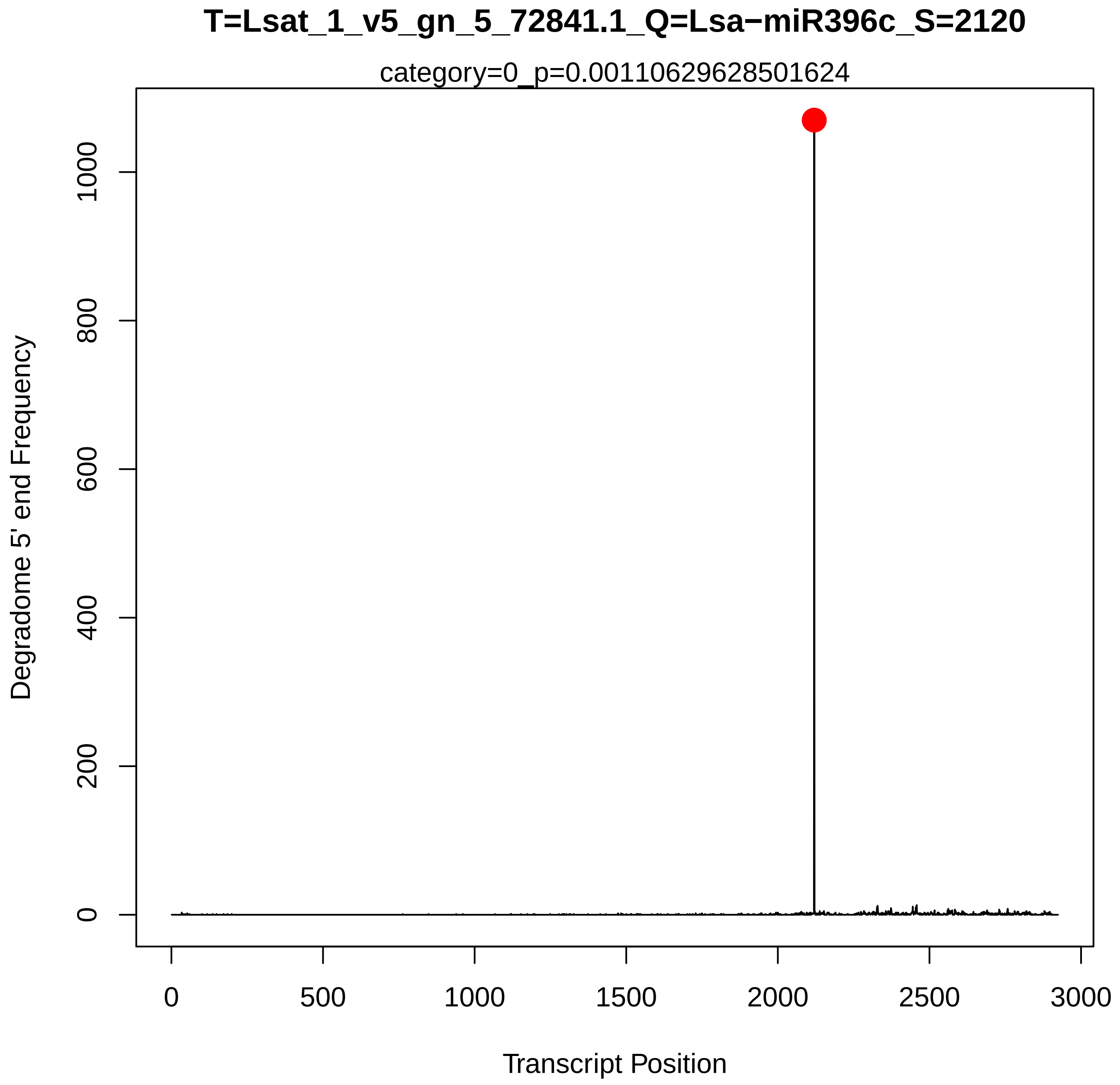
T=Lsat_1_v5_gn_5_72841.1_Q=Lsa−miR396c_S=2120
category=0_p=0.00110629628501624
Degradome 5' end Frequency
0
200
400
600
800
1000
0
500
1000
1500
2000
2500
3000
Transcript Position
